# Associations of Cardiometabolic Indices With Peptides Related to Hypertensive Disorders of Pregnancy in Adult Men

**DOI:** 10.7759/cureus.62232

**Published:** 2024-06-12

**Authors:** Ichiro Wakabayashi, Mitsuaki Yanagida, Yoshihiko Araki

**Affiliations:** 1 Department of Environmental and Preventive Medicine, Hyogo Medical University, School of Medicine, Nishinomiya, JPN; 2 Institute for Environmental and Gender-Specific Medicine, Juntendo University Graduate School of Medicine, Urayasu, JPN; 3 Department of Pathology and Microbiology, Division of Microbiology, Nihon University School of Medicine, Tokyo, JPN

**Keywords:** peptide biomarker, lipid accumulation product, hypertensive disorders of pregnancy, hematometabolic index, cardiovascular risk

## Abstract

Background: Seven peptides with low molecular weights in blood have been identified as possible biomarkers of hypertensive disorders of pregnancy (HDP). A history of HDP is known to be associated with a high risk of cardiovascular disease in the later life of women with HDP. However, it remains to be determined whether HDP-related peptides are useful biomarkers of cardiovascular disease in the general population. The purpose of this study was to determine the relationships between these peptides and cardiometabolic risk in adult men.

Methods: We investigated the relationships between HDP-related peptides and two recent indices of cardiometabolic risk, hematometabolic index (HMI) and lipid accumulation product (LAP), in male workers aged 35 to 69 years. Concentrations of the HDP-related seven peptides with mass/charge ratios (*m/z*) of 2081 (P-2081), 2091 (P-2091), 2127 (P-2127), 2209 (P-2209), 2378 (P-2378), 2858 (P-2858), and 3156 (P-3156) were measured simultaneously by using a mass spectrometer. Standardized partial regression coefficients (β) were obtained in multivariable linear regression analysis, and mean levels of the log-transformed HMI and LAP were compared in tertile groups of each peptide in the analysis of covariance with adjustment for age, habits of smoking and alcohol drinking, history of diabetes, and medication therapy for dyslipidemia.

Results: There was a significant positive correlation between the HMI and the serum level of P-2378 (β = 0.310), a fragment of complement component 4, while a significant inverse correlation (β = -0.389) was obtained between the LAP and the serum level of P-3156, a fragment of inter-α-trypsin inhibitor heavy chain H4. Other peptides (P-2081, P-2091, P-2127, P-2209, and P-2858) did not show significant correlations with the HMI or LAP. The log-transformed HMI tended to be higher with an increase in the tertile for P-2378. The mean level of log-transformed LAP in the first tertile group of P-3156 was significantly higher than those in the second and third tertile groups of P-3156.

Conclusion: The HDP-related peptides with *m/z* of 2378 and *m/z* of 3156 were shown to be associated with the HMI and LAP, respectively, which are recent indices reflecting cardiometabolic risk. Therefore, the peptides with *m/z* of 2378 and *m/z* of 3156 were thought to be potential biomarkers for discrimination of cardiovascular risk in adult men. Further studies on the relationships between the peptides and cardiovascular risk factors in non-pregnant women are needed to confirm the findings of this study.

## Introduction

Hypertensive disorders of pregnancy (HDP) are serious complications that determine the prognosis of pregnancy [1]. It is also known that the risk of cardiovascular disease in the later life of patients with HDP is higher than that in women with a normal pregnancy not complicated by HDP [2,3]. We identified seven HDP-associated peptides with low molecular weights in serum and proposed that they are potential biomarkers for early prevention of HDP [4]. These peptide fragments were found to originate from fibrinogen-α (P-2091), kininogen (P-2081, P-2127, P-2209), complement C4 (P-2378), α-2-HS-glycoprotein (P-2858), and inter-α-trypsin inhibitor heavy chain H4 (P-3156), all of which were named on the basis of their mass/charge ratios (m/z) as shown above [4]. These peptides do not appear to have disease-related hemodynamic changes that are limited to pregnant women. That is, some of the HDP-related peptides were shown to be linked to risk factors for atherosclerotic diseases including blood pressure, body mass index, and blood lipids (triglycerides and HDL cholesterol) in healthy men, and further, to the degree of leg ischemia in male and female patients with lower extremity artery disease (LEAD) [5,6].

Recently, we have proposed the hematometabolic index (HMI), which is calculated as a product of hemoglobin concentration and leukocyte count after modifications, as a new biomarker for discriminating cardiovascular risks, such as metabolic syndrome [7]. Leukocyte count in peripheral blood has been shown to be a predictor of atherosclerotic cardiovascular disease [8-12]. Polycythemia has also been known to increase the risk of cardiovascular events [13,14]. Therefore, the HMI is a reasonable marker for predicting the cardiovascular risk. Thus, in addition to the classical cardiovascular risk factors, hemoglobin concentration and leukocyte count have been reported to be potential cardiovascular risks and they comprise a new index called HMI. Given this line of research, it would be interesting to know the relationships between the HMI and the above peptides initially identified as HDP-related factors, especially in a general population. Therefore, the purpose of this concise study was to investigate relationships between the HMI and HDP-related peptides in adult male workers. We also analyzed correlations between the peptides and another recent cardiometabolic index, lipid accumulation product (LAP), reflecting adiposity (waist circumference), and blood lipids (triglycerides) [15]. The LAP has been reported to be associated with the risk of cardiovascular disease [16-20] and is a reasonable index for predicting cardiovascular risk since waist circumference and triglycerides are known to be associated with an increase in the risk of cardiovascular events [21-24].

## Materials and methods

Subjects

The cohort of this study involved 51 adult male workers (35~69 years old; mean age: 53.4 years) receiving annual health checkup examinations in a construction company in the Yamagata Prefecture, Japan. Almost all of the men working at this company participated in the present study after written informed consent. The participants were thought not to be exposed to environments influencing blood levels of the HDP-related peptides in their workplaces. This study is a pilot study on the relationships between the new cardiometabolic indices and the HDP-related peptides. The sample size of this study was determined by reference to our former study on the relationships between the HDP-related peptides and LEAD [6]. The protocol of this cross-sectional study was approved by the Hyogo College of Medicine Ethics Committee (No. 3449) on 18th February 2021. Written informed consent was obtained from all of the participants. Histories of alcohol consumption, cigarette smoking, illness, and therapy(ies) for illness(es) were surveyed by self-reported questionnaires as described previously [5]. Habitual smoking and alcohol drinking were evaluated by the amount of daily average cigarette consumption and frequency of weekly alcohol drinking, respectively. Smokers were categorized as light smokers (20 cigarettes or less), heavy smokers (between 21 and 40 cigarettes), and very heavy smokers (more than 40 cigarettes). The frequency of individual alcohol drinking was asked as “How frequently do you drink alcohol?”, and drinkers were categorized as nondrinkers (“never”), occasional drinkers (“sometimes”), and regular drinkers (“every day”). Regarding the HMI, we used the following exclusion criteria for subjects showing low hemoglobin concentrations (13 g/dL or lower) or low leukocyte counts (3000/μL or lower). One subject was excluded from the analysis because of his low hemoglobin level (< 13 g/dl).

Measurements

At the health checkup examination, height was measured with light clothes and waist circumference was measured at the navel level according to the definition of the Japanese Committee for the Diagnostic Criteria of Metabolic Syndrome [25]. Diabetes was diagnosed in subjects showing hemoglobin A_1c_ levels of 6.5% or higher [26] and/or having a history of medication therapy for diabetes. Venous blood was collected from each participant in the morning after overnight fasting, and serum was separated from the blood by centrifugation. Hemoglobin, leukocyte count, and hemoglobin A_1c_ in whole blood and triglycerides in serum were measured as described previously [7]. The HMI and LAP were calculated as follows: HMI = (hemoglobin [g/dl] - 13.0) x (leukocyte count [/μL] - 3000); LAP = triglycerides (mM) x (waist circumference [cm] - 65) [7,15].

Concentrations of the seven HDP-related peptides in serum were determined by mass spectrophotometry as described previously [5]. Briefly, each serum sample was spiked with stable isotope-labeled (SI) internal standard peptides, and the peptide fraction was prepared with a graphite carbon tip device. The seven target peptides were quantified with an LC-MS/MS system using the multiple reaction monitoring mode. The concentration of each peptide in serum was calculated by the ratio of the peak areas of the natural and internal standard SI peptides.

Statistical analysis

Statistical analyses were performed using a computer software program (IBM SPSS Statistics for Windows, Version 25, Released 2017; IBM Corp., Armonk, New York, United States). Categorical variables are presented as percentage. Continuous variables were summarized as means with standard deviations or standard errors or medians with interquartile ranges, as appropriate. Since the seven peptide levels, HMI, LAP, or triglycerides did not show normal distributions, they were used for analyses after base-10 logarithmic transformation. Pearson’s correlation coefficients and standardized partial regression coefficients (β) were calculated in univariable and multivariable linear regression analyses, respectively. The values of concentrations of each peptide in subjects were arranged in ascending order, and then the subjects were divided into three tertile groups of approximately equal sizes. In tertile groups of each peptide, mean levels of the HMI and LAP after logarithmic transformation were compared by using ANOVA followed by Scheffé’s F-test as a post hoc test in univariable analysis and ANCOVA followed by Student’s t-test after Bonferroni correction in multivariable analysis. In multivariable analysis, age, habits of smoking and alcohol drinking, and histories of diabetes and medication therapy for dyslipidemia were used as other explanatory variables and covariates. The trends of HMI and LAP levels in three tertiles of each peptide were analyzed by using the Jonckheere-Terpstra test. When the probability (*p*) value is less than 0.05, it is judged as significant.

## Results

The characteristics of the subjects are shown in Table 1. Forty-five percent of the subjects were smokers and 86% of the subjects were occasional or regular drinkers. The percentages of subjects with histories of diabetes and anti-dyslipidemic drug therapy were relatively low (2.0% and 7.8%, respectively). There was a large range of concentrations of the seven peptides in serum: The concentrations of P-2858 were relatively high (> 1 μg/ml) and those of P-2081, P-2127, and P-2209 were relatively low (< 1.0 ng/ml). Cut-off values of the HMI and LAP for men have been proposed to be 9850 and 37.2, respectively [7,27], and according to these values, 31.4% and 27.5% of the subjects showed high levels of the HMI and LAP, respectively.

**Table 1:** Characteristics of the participants. Shown are number, percentage, mean with standard deviation, or median with interquartile range in parenthesis for each variable. HMI, hematometabolic index; LAP, lipid accumulation product.

Variable	Number, percentage, mean, or median
Number	51
Age (years)	53.4 ± 9.1
Smokers (%)	
Light	35.3
Heavy	9.8
Alcohol drinkers (%)	
Occasional	19.6
Regular	66.7
Therapy for dyslipidemia (%)	7.8
Diabetes (%)	2.0
Height (cm)	170.8 ± 5.5
Waist circumference (cm)	84.2 ± 8.2
Hemoglobin (g/dl)	15.3 ± 0.9
Leukocyte count (/μl)	6298 ± 1645
Triglycerides (mg/dl)	123 (84, 190)
HMI	7000 (2800, 10530)
LAP	29.2 (12.5, 38.3)
Hemoglobin A_1c_ (%)	5.51 ± 0.35
P-2081 (ng/ml)	0.14 (0.10, 0.17)
P-2091 (ng/ml)	31.5 (28.2, 38.0)
P-2127 (ng/ml)	0.05 (0.04, 0.06)
P-2209 (ng/ml)	0.35 (0.27, 0.40)
P-2378 (ng/ml)	102.6 (88.3, 119.0)
P-2858 (ng/ml)	3158 (2903, 3874)
P-3156 (ng/ml)	3.98 (3.07, 5.13)

The results for correlations of each peptide concentration with the HMI and LAP are shown in Table 2. There were significant correlations between the HMI and LAP in univariable analysis and multivariable analysis. In both univariable analysis and multivariable analysis, P-3156 showed significant inverse correlations with the LAP but not with the HMI. P-2378 showed a marginally significant positive correlation with the HMI in univariable analysis and a significant positive correlation with the HMI in multivariable analysis, while there was no significant correlation between P-2378 and LAP in either univariable analysis or multivariable analysis. P-2081 and P-2091 showed marginally significant correlations with the LAP and HMI, respectively, in univariable analysis, but those correlations were not significant in multivariable analysis. Except for P-2378 and P-3156, there were no HDP-related peptides that showed significant correlations with the HMI or LAP.

**Table 2:** Correlations of the HDP-related peptides with the HMI and LAP. Shown are Pearson’s correlation coefficients in univariable analysis (univar) and standardized partial regression coefficients in multivariable analysis (multivar). Each variable was used for analysis after base-10 logarithmic transformation. In multivariable analysis, age, habits of smoking and alcohol drinking, and histories of diabetes and therapy for dyslipidemia were adjusted. Symbols indicate significant correlations (*, *p* < 0.05; **, *p* < 0.01) and marginally significant correlations (#, *p* = 0.071; ##, *p* = 0.060; ###, *p* = 0.075). HMI, hematometabolic index; LAP, lipid accumulation product.

	HMI (univar)	HMI (multivar)	LAP (univar)	LAP (multivar)
HMI	—	—	0.324*	0.309*
LAP	0.324*	0.345*	—	—
P-2081	0.025	-0.009	-0.254^###^	-0.210
P-2091	-0.255^#^	-0.179	-0.100	-0.065
P-2127	0.130	0.117	0.000	0.029
P-2209	-0.158	-0.170	-0.171	-0.057
P-2378	0.265^##^	0.310*	-0.093	-0.086
P-2858	-0.136	-0.134	0.002	0.117
P-3156	-0.118	-0.073	-0.427**	-0.389**

**Figure 1:**
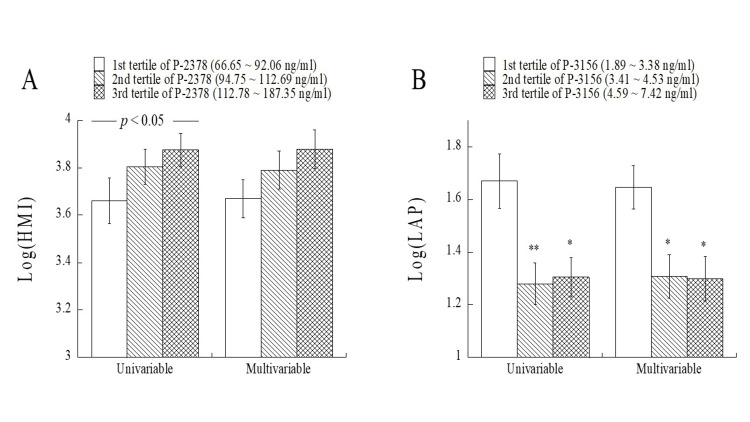
Comparisons of the mean log-transformed HMI and log-transformed LAP in the tertile groups of P-2378 and P-3156, respectively. Log-transformed HMI and LAP levels are shown as means with standard errors. In multivariable analysis, age, habits of smoking and alcohol drinking, and histories of diabetes and therapy for dyslipidemia were adjusted. There was a significant increasing trend (p < 0.05) of HMI with an increase in the tertile for P-2378. Asterisks indicate significant differences from the first tertile group of P-3156 (*, *p* < 0.05; **, *p* < 0.01). HMI, hemato-metabolic index; LAP, lipid accumulation product.

The mean levels of the log-transformed HMI and log-transformed LAP were compared among the tertile groups of P-2378 and P-3156, respectively (Figure 1). The log-transformed HMI tended to be higher with an increase in the tertile for P-2378 in univariable and multivariable analyses. There was a significant increasing trend (*p* < 0.05 by the Jonckheere-Terpstra test) of the HMI with an increase in the tertile for P-2378 in univariable analysis. The mean levels of the log-transformed LAP in the first tertile group of P-3156 were significantly higher than those in the second and third tertile groups of P-3156 in univariable and multivariable analyses.

The correlations of P-2378 and P-3156 with the components of the HMI and LAP were investigated (Table 3). In univariable and multivariable analyses, P-3156 showed significant correlations with waist circumference and triglycerides but not with hemoglobin and leukocyte count. P-2378 did not show significant correlations with hemoglobin or leukocyte count. There were also no significant correlations of P-2378 with waist circumference or triglycerides in the univariable and multivariable analyses.

**Table 3:** Correlations of P-2378 and P-3156 with the components of the HMI and LAP. Shown are Pearson’s correlation coefficients in univariable analysis (univar) and standardized partial regression coefficients in multivariable analysis (multivar). Levels of P-2378, P-3156, and triglycerides were used for analysis after base-10 logarithmic transformation. In multivariable analysis, age, habits of smoking and alcohol drinking, and histories of diabetes and therapy for dyslipidemia were adjusted. Symbols indicate significant correlations (*, *p* < 0.05; **, *p* < 0.01). HMI, hematometabolic index; LAP, lipid accumulation product.

	P-2378 (univar)	P-2378 (multivar)	P-3156 (univar)	P-3156 (multivar)
Hemoglobin	0.142	0.151	-0.011	0.050
Leukocyte count	0.193	0.239	-0.143	-0.123
Waist circumference	0.073	0.046	-0.372**	-0.361**
Triglycerides	-0.176	-0.168	-0.375**	-0.312*

## Discussion

This study for the first time showed relationships between HDP-related peptides and indices of cardiometabolic risk including the HMI and LAP in adult men. There was a significant positive correlation between the HMI and LAP, which may be explained by the fact that both reflect cardiometabolic risk [7,27,28]. Among the seven HDP-related peptides, only P-2378 and P-3156 were associated with the HMI and LAP, respectively. P-2378 and P-3156 are fragments of complement C4 and inter-α-trypsin inhibitor heavy chain H4, respectively. Thus, different HDP-related peptides showed associations with different indices of cardiometabolic risk. One possible explanation for these differences is the involvement of different proteases in in vivo production of P-2378 and P-3156, which needs to be elucidated in future studies. Moreover, relationships of the above parent proteins of these peptides with HMI and LAP remain to be clarified.

P-3156 was inversely correlated with waist circumference and triglycerides (Table 3). Therefore, it is reasonable that P-3156 was inversely correlated with LAP, which is an index calculated from waist circumference and triglycerides [15]. P-2378 did not show significant correlations with waist circumference or triglycerides (Table 3), which agrees with the finding that there was no significant correlation between P-2378 and LAP (Table 2).

Among the seven HDP-related peptides, P-2378 is the only peptide that showed a significant correlation with the HMI, which is calculated by hemoglobin and leukocyte count. However, P-2378 did not show significant correlations with hemoglobin alone or leukocyte count alone (Table 3). Thus, the HMI is a more sensitive biomarker for cardiometabolic risk than hemoglobin alone and leukocyte count alone. The present study is the first study in which the HMI is suggested to be a clinically useful biomarker. The six HDP-related peptides other than P-2378 did not show significant correlations with hemoglobin alone or leukocyte count alone (data not shown). In patients with LEAD, P-2378 but not P-3156 has been reported to show an association with the degree of leg ischemia due to atherosclerosis evaluated by the ankle-brachial arterial index [6]. Therefore, P-2378, a fragment of complement C4, is suggested to be a potential biomarker for cardiometabolic risk in the general population as well as for leg ischemia in patients with LEAD. It is a new concept that a fragment of complement is a biomarker for cardiovascular disease. Thus, future studies on fragments of known proteins in relation to various diseases are warranted.

There are limitations to this study. The number of subjects in this pilot study was small, and further studies using a database of a larger population are needed to confirm the findings of this study. Although age, habits of smoking and alcohol drinking, and histories of diabetes and medication therapy for dyslipidemia were adjusted in the multivariable analysis, there are other possible confounding factors, e.g., physical activity, nutrition, and socio-economic status, information of which was not available in the present study. Since this study is cross-sectional in its design, further prospective studies and randomized control trials are also needed to clarify causal relationships between peptides and cardiovascular risk in the future. The mechanisms for the associations between the new peptides and cardiovascular risk also need to be clarified in future studies. Since the seven HDP-related peptides were originally proposed for early diagnosis of HDP in pregnant women, it would be interesting to know the significance of these peptides for the prevention of cardiovascular disease in women. However, the subjects of the present study were all males and, moreover, there has been no study in which the relationships between the HDP-related peptides and cardiovascular risk were investigated in non-pregnant women. Further studies are therefore needed to clarify the significance of these peptides for the prevention of cardiovascular disease in women.

## Conclusions

In adult men, the HDP-related peptides with *m/z* of 2378 and *m/z* of 3156 were shown to be associated with the HMI and LAP, respectively, which are recent indices reflecting cardiometabolic risk. Therefore, these peptides might be potential biomarkers for the discrimination of high risk for future cardiovascular events; however, more research on the peptides is needed to determine their roles as biomarkers. Further studies using female subject groups and adjusting more confounding factors are also needed to confirm the findings of this study.
